# Development and validation of novel machine learning-based prognostic models and propensity score matching for comparison of surgical approaches in mucinous breast cancer

**DOI:** 10.3389/fendo.2025.1557858

**Published:** 2025-06-03

**Authors:** Chunmei Chen, Jundong Wu, Yutong Fang, Yong Li, Qunchen Zhang

**Affiliations:** ^1^ Department of Breast, Jiangmen Central Hospital, Jiangmen, Guangdong, China; ^2^ The Breast Center, Cancer Hospital of Shantou University Medical College, Shantou, Guangdong, China

**Keywords:** mucinous breast cancer, machine learning, prognosis, surgery, propensity score matching

## Abstract

Mucinous breast cancer (MBC) is a rare subtype of breast cancer with specific clinicopathologic and molecular features. Despite MBC patients generally having a favorable survival prognosis, there is a notable absence of clinically accurate predictive models. Patients diagnosed with MBC from the SEER database spanning 2010 to 2020 were included for analysis. Cox regression analysis was conducted to identify independent prognostic factors. Ten machine learning algorithms were utilized to develop prognostic models, which were further validated using MBC patients from two Chinese hospitals. Cox analysis and propensity score matching were applied to evaluate survival differences between MBC patients undergoing mastectomy and breast-conserving surgery (BCS). We determined that the XGBoost models were the optimal models for predicting overall survival (OS) and breast cancer-specific survival (BCSS) in MBC patients with the most accurate performance (AUC=0.833-0.948). Moreover, the XGBoost models still demonstrated robust performance in the external test set (AUC=0.856-0.911). Patients treated with BCS exhibited superior OS compared to those undergoing mastectomy (p < 0.001, HR: 0.60, 95% CI: 0.47-0.77). However, no significant difference was observed in the risk of breast cancer-related mortality. We have successfully developed 6 optimal prognostic models utilizing the XGBoost algorithm to accurately predict the survival of MBC patients. We also developed an interactive web application to facilitate the utilization of our models by clinicians or researchers. Notably, we observed a significant improvement in OS for patients undergoing BCS.

## Introduction

Mucinous breast cancer (MBC) is a rare histological subtype of breast cancer (BC), constituting approximately 2–5% of all BC cases (1). Despite its low incidence, the global rise in BC prevalence has led to a proportional increase in MBC diagnoses (2, 3). Compared to more common BC subtypes, such as infiltrating ductal carcinoma (IDC), MBC exhibits distinct clinicopathologic and molecular characteristics, including a higher prevalence of hormone receptor expression and a lower propensity for lymph node metastasis (4–8). MBC predominantly affects postmenopausal women and is generally associated with a favorable prognosis (9, 10). Given the scarcity of clinical data, systemic treatment strategies for MBC largely derive from therapeutic approaches established for IDC (11, 12).

Several nomograms have been developed to predict early-stage MBC prognosis (13–15). However, due to the rarity of MBC, these models have been constructed exclusively using data from the Surveillance, Epidemiology, and End Results (SEER) database, without external validation to assess their generalizability. Furthermore, their predictive performance remains suboptimal, with area under the curve (AUC) values or concordance indices (C-index) ranging from 0.7 to 0.8. Machine learning (ML), an advancing field in medicine, offers a robust framework of algorithms capable of data representation, adaptation, learning, prediction, and analysis (16–18). Deep neural networks have been employed to support surgical decision-making and survival prediction in patients with *de novo* metastatic BC (17). Extreme gradient boosting (XGBoost), an optimized gradient boosting tree algorithm, refines predictive accuracy by iteratively updating model parameters through the negative gradient of the loss function, enabling its predictions to converge progressively toward true values (19). XGBoost has gained traction in medical research for disease prediction, diagnostic support, and risk assessment. Li et al. developed high-performance XGBoost-based prognostic models for advanced BC (20, 21), achieving AUC values of 0.821 to 0.910 in patients with PR-positive BC (22). Additionally, XGBoost models have demonstrated reliable predictive accuracy for survival outcomes in patients with second primary BC, with AUC values between 0.817 and 0.825 (23). Despite these advances, XGBoost has yet to be applied in MBC prognosis prediction.

The treatment of MBC remains unsupported by robust evidence and standardized guidelines. Currently, mastectomy and breast-conserving surgery (BCS) represent the primary surgical interventions for MBC. Observational studies suggest that BCS may confer a prognostic advantage over mastectomy (24). However, the inherent limitations of retrospective observational studies, particularly selection bias due to the absence of randomized allocation, undermine the reliability of these findings. Propensity score matching (PSM) is frequently employed to balance covariates between study and control groups, thereby reducing potential confounding factors. However, the survival advantage of specific surgical approaches for MBC has yet to be definitively established following PSM.

This study constructed predictive models for overall survival (OS) and breast cancer-specific survival (BCSS) in patients with MBC using ten ML algorithms trained on the SEER database. Additionally, retrospective clinical data from patients with MBC in two Chinese hospitals were incorporated to evaluate the models’ generalizability. PSM was further applied to assess survival outcomes between patients undergoing mastectomy and those undergoing BCS. The findings aim to enhance prognostic assessment and inform personalized treatment strategies for MBC through the identification of an optimal predictive model.

## Materials and methods

### Patients and study design

**Figure 1:**
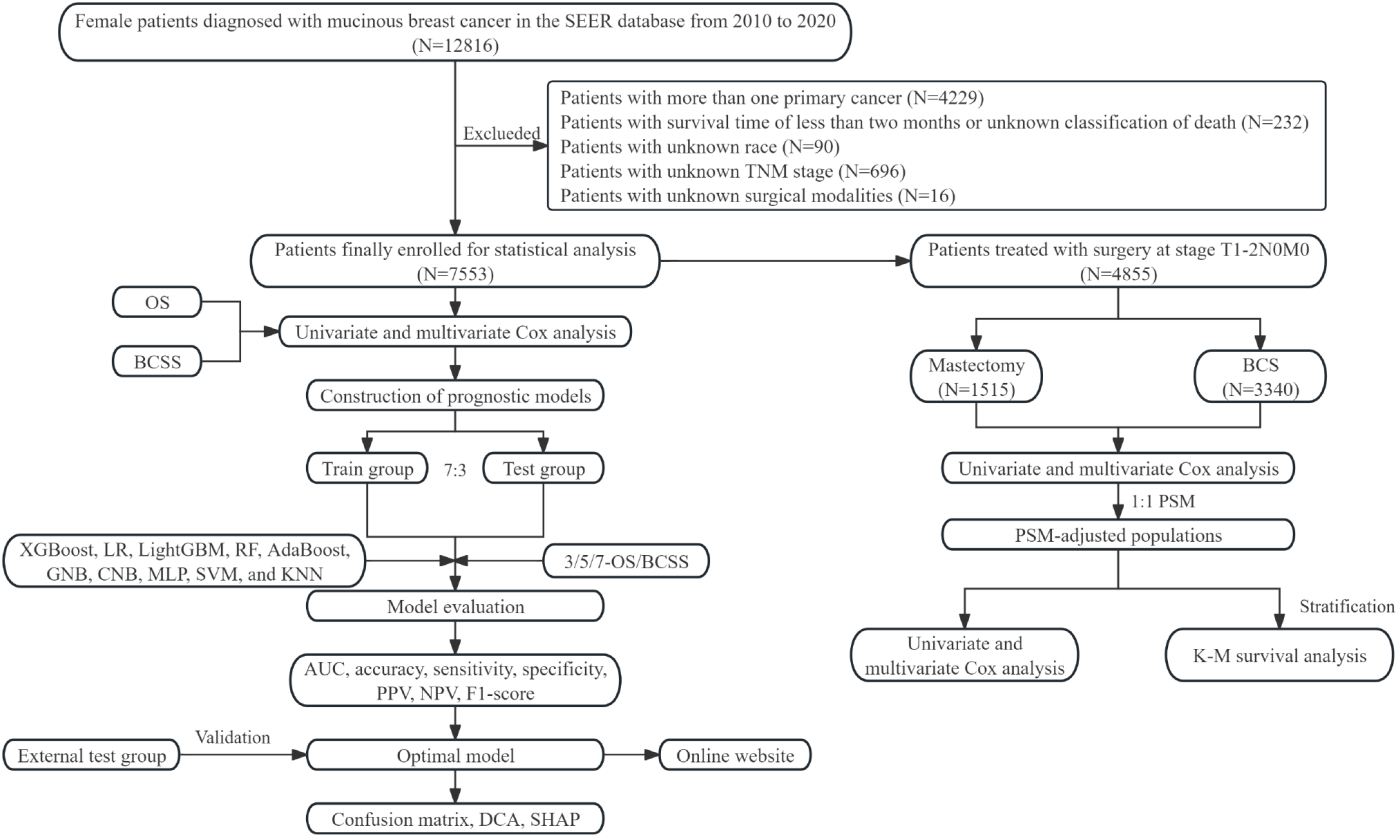
Flow chart of this study. SEER, surveillance, epidemiology, and end results; OS, overall survival; BCSS, breast cancer-specific survival; XGBoost, extreme gradient boosting; LR, logistic regression; LightGBM, light gradient boosting machine; RF, random forest; AdaBoost, adaptive boosting; GNB, gaussian naive bayes; CNB, complement naive bayes; MLP, multi-layer perceptron neural networks; SVM, support vector machine; KNN, k-nearest neighbors; AUC, area under the curve; PPV, positive predictive value; NPV, negative predictive value; DCA, decision curve analysis; SHAP, SHapley Additive exPlanations; BCS, breast-conserving surgery; K-M, Kaplan-Meier.

The study design is illustrated in the flowchart (**Figure 1**). Patient data were obtained from three sources. The SEER database, a publicly available resource curated by the National Cancer Institute, provided the primary dataset. Specifically, SEER 17 registries research data [(2000–2020); version 8.4.2] were utilized, with the following inclusion criteria: (1) female sex, (2) diagnosis between 2010 and 2020, (3) histological classification of ICD-O-3 8480/3, (4) complete clinical information, and (5) survival duration exceeding one month. Patients with multiple primary tumors were excluded. Additionally, retrospective data were collected from patients with MBC treated at Jiangmen Central Hospital (JCH) (n=98) and the Cancer Hospital of Shantou University Medical College (CHSU) (n=85) between January 2010 and October 2020, adhering to the same inclusion criteria. Ethical approval was granted by the respective institutional review boards of JCH (No. 2023146) and CHSU (No. 2023130).

### Data collection

Collected patient variables included age, race, marital status, median household income, tumor location, histologic grade, molecular subtype, T stage, N stage, M stage, surgical intervention, radiotherapy, and chemotherapy. The primary endpoint was OS, while BCSS served as the secondary endpoint. The median follow-up time was 60 months (58.6-61.4) for patients from the SEER database and 80 months (73.1-87.0) for patients from two hospitals in China.

### Feature selection, model construction, and evaluation

To eliminate redundant variables, univariate and multivariate Cox regression analyses were conducted to identify independent prognostic factors. Statistically significant variables were incorporated as features in ML model development. Prognostic models for OS and BCSS at 3, 5, and 7 years were constructed using ten widely applied ML algorithms: XGBoost, logistic regression (LR), light gradient boosting machine (LightGBM), random forest (RF), adaptive boosting (AdaBoost), Gaussian naive Bayes (GNB), complement naive Bayes (CNB), multi-layer perceptron neural networks (MLP), support vector machine (SVM), and k-nearest neighbors (KNN). To enhance model robustness, ten-fold cross-validation and grid search optimization were employed to fine-tune hyperparameters. Patients from the SEER database were randomly divided into training and internal test cohorts at a 7:3 ratio, while two independent Chinese hospital cohorts served as external validation datasets to assess model generalizability.

Model performance was evaluated using the AUC (25), accuracy, sensitivity, specificity, positive predictive value (PPV), negative predictive value (NPV), and F1 score. A confusion matrix was used to visualize classification accuracy, while decision curve analysis (DCA) assessed the clinical utility of the models. Feature importance was quantified using SHapley Additive exPlanations (SHAP) values, computed *via* the “shap” package.

To facilitate clinical application, an interactive web-based platform was developed using the Streamlit framework, providing access to the optimized predictive models for real-time use by clinicians.

### PSM

To further evaluate the prognostic impact of mastectomy versus BCS in patients with MBC, a cohort of 5,760 patients was extracted from the SEER database. Inclusion criteria were: (1) stage T1-2N0M0 disease and (2) receipt of either mastectomy or BCS. Exclusion criteria included: (1) mastectomy with adjuvant radiotherapy and (2) BCS without radiotherapy. To mitigate confounding bias inherent in retrospective studies, 1:1 PSM was conducted based on the ML model’s selected features to balance baseline characteristics between surgical groups.

Univariate and multivariate Cox regression analyses were performed before and after PSM to assess survival outcomes. Additionally, a forest plot was used to visualize survival differences across various subgroups of patients with MBC within the PSM-adjusted cohort.

### Statistical analysis

Cox regression analyses were further employed to identify key prognostic features for model construction. Statistical analyses were conducted using R software (version 4.2.1, r-project.org/) and Python (version 3.8, Python Software Foundation). Statistical significance was defined as P < 0.05.

## Results

### Clinicopathologic characteristics

A total of 7,553 eligible patients with MBC were identified from the SEER database. As summarized in **Table 1**, 16.64% (1,257) were ≤ 50 years old, 29.82% (2,252) were between 51 and 65 years old, and 53.54% (4,044) were ≥ 66 years old. The majority of patients were White (74.22%), and nearly half were married (49.36%), while 16.62% were single or identified as homosexual. In terms of socioeconomic status, 75.73% had a median household income exceeding $60,000. Tumors were most frequently located in the upper outer quadrant (25.27%), followed by the lower inner quadrant (10.31%), lower outer quadrant (9.02%), and central quadrant (6.88%). Grade I tumors accounted for 54.84% of cases, whereas Grades III and IV were observed in only 8.78% of patients. The HR+/HER2− subtype was predominant, comprising 94.03% of cases. The distribution of tumor stages showed that T1, T2, T3, and T4 tumors accounted for 63.55%, 29.55%, 5.20%, and 1.69% of cases, respectively. Nodal involvement was minimal, with 90.67% classified as N0, while N1, N2, and N3 stages comprised 7.70%, 0.98%, and 0.65% of cases, respectively. Distant metastases (M1) were present in only 1.22% of patients. Regarding treatment, 94.55% underwent mastectomy or BCS, 51.95% received radiotherapy, and 12.41% received chemotherapy. Correlation analysis between variables demonstrated no evidence of multicollinearity, as visualized in the heatmap (**Supplementary Figure S1**).

**Table 1:** Baseline characteristics of patients with mucinous breast cancer in the SEER database.

Characteristic	Variables	Cases	%
Age	≤50	1257	16.64
	51-65	2252	29.82
	≥66	4044	53.54
Race	White	5606	74.22
	Black	901	11.93
	Others	1046	13.85
Marital status	Singled/homosexual	1255	16.62
	Married	3728	49.36
	Widow/divorced/others	2570	34.03
Median household income (inflation adjusted)	<$40,000	208	2.75
	$40,00-59,999	1625	21.52
	$60,000+	5720	75.73
Tumor location	Upper outer	1909	25.27
	Lower outer	779	10.31
	Lower inner	681	9.02
	Upper inner	1026	13.58
	Central	520	6.88
	Others	2638	34.93
Grade	Well differentiated	4142	54.84
	Moderate differentiated	2748	36.38
	Poorly differentiated	193	2.56
	Unknown	470	6.22
Subtype	HR+/HER2+	370	4.9
	HR+/HER2-	7102	94.03
	HR-/HER2+	50	0.66
	HR-/HER2-	31	0.41
T stage	T1	4800	63.55
	T2	2232	29.55
	T3	393	5.20
	T4	128	1.69
N stage	N0	6848	90.67
	N1	582	7.70
	N2	74	0.98
	N3	49	0.65
M stage	M0	7461	98.78
	M1	92	1.22
Surgery	No	336	4.45
	Mastectomy	2129	28.19
	Breast-conserving surgery	5088	67.36
Radiotherapy	No/unknown	3629	48.05
	Yes	3924	51.95
Chemotherapy	No/unknown	6616	87.59
	Yes	937	12.41

### Feature selection

Univariate Cox regression analysis (**Table 2**) identified age, race, marital status, median household income, subtype, T stage, N stage, M stage, surgery, radiotherapy, and chemotherapy as significant prognostic factors for OS. Similarly, BCSS was significantly influenced by age, race, marital status, median household income, histologic grade, subtype, T stage, N stage, M stage, surgery, radiotherapy, and chemotherapy.

**Table 2:** Univariate and multivariate Cox analyses of patients with mucinous breast cancer in the SEER database.

Variables	Univariate Cox analysis						Multivariate Cox analysis					
	OS			BCSS			OS			BCSS		
	HR	95%CI	P	HR	95%CI	P	HR	95%CI	P	HR	95%CI	P
Age												
≤50	Reference			Reference			Reference			Reference		
51-65	1.70	1.18-2.44	0.004	0.80	0.48-1.34	0.402	1.91	1.32-2.78	0.001	1.02	0.59-1.76	0.954
66+	7.01	5.08-9.67	<0.001	1.64	1.06-2.52	0.025	6.36	4.50-8.99	<0.001	2.46	1.45-4.19	0.001
Race												
White	Reference			Reference			Reference			Reference		
Black	1.07	0.88-1.30	0.485	1.59	1.08-2.34	0.018	0.96	0.78-1.17	0.683	1.11	0.73-1.68	0.622
Others	0.57	0.45-0.72	0.001	0.55	0.31-0.98	0.041	0.78	0.61-1.00	0.054	0.79	0.44-1.42	0.432
Marital status												
Singled/homosexual	Reference			Reference			Reference			Reference		
Married	0.67	0.54-0.83	<0.001	0.45	0.29-0.69	<0.001	0.61	0.49-0.76	<0.001	0.60	0.38-0.95	0.029
Widow/divorced/others	1.98	1.63-2.40	<0.001	1.26	0.85-1.85	0.247	1.05	0.86-1.29	0.632	1.04	0.67-1.61	0.857
Median household income (inflation adjusted)												
<$40,000	Reference			Reference			Reference			Reference		
$40,00-59,999	0.81	0.57-1.13	0.215	0.58	0.30-1.15	0.121	0.76	0.54-1.07	0.115	0.44	0.22-0.88	0.020
$60,000+	0.61	0.44-0.85	0.003	0.45	0.24-0.86	0.016	0.65	0.46-0.90	0.011	0.33	0.17-0.64	0.001
Tumor location												
Upper outer	Reference			Reference			Reference			Reference		
Lower outer	0.88	0.68-1.14	0.330	0.81	0.45-1.44	0.470	/	/	/	/	/	/
Lower inner	0.93	0.72-1.21	0.578	0.75	0.40-1.39	0.359	/	/	/	/	/	/
Upper inner	1.05	0.84-1.31	0.663	1.10	0.69-1.773	0.688	/	/	/	/	/	/
Central	1.15	0.88-1.51	0.317	0.72	0.35-1.46	0.360	/	/	/	/	/	/
Others	1.15	0.97-1.36	0.104	1.04	0.72-1.51	0.836	/	/	/	/	/	/
Grade												
Well differentiated	Reference			Reference			Reference			Reference		
Moderate differentiated	0.92	0.79-1.06	0.241	1.49	1.07-2.09	0.018	/	/	/	1.48	1.05-2.08	0.026
Poorly differentiated	0.96	0.63-1.45	0.832	3.20	1.70-6.04	<0.001	/	/	/	2.06	1.02-4.18	0.045
Unknown	1.13	0.91-1.41	0.273	2.51	1.62-3.89	<0.001	/	/	/	1.08	0.67~1.74	0.745
Subtype												
HR+/HER2+	Reference			Reference			Reference			Reference		
HR+/HER2-	1.49	1.05-2.12	0.027	0.79	0.43-1.46	0.453	0.84	0.58-1.22	0.358	1.06	0.55-2.05	0.854
HR-/HER2+	1.22	0.48-3.14	0.677	1.41	0.31-6.36	0.656	1.26	0.48-3.26	0.640	0.80	0.16-3.94	0.781
HR-/HER2-	3.37	1.55-7.31	0.002	4.78	1.52-15.01	0.007	1.77	0.81-3.86	0.154	4.58	1.38-15.17	0.013
T stage												
T1	Reference			Reference			Reference			Reference		
T2	1.76	1.52-2.03	<0.001	3.03	2.10-4.35	<0.001	1.78	1.53-2.07	<0.001	2.14	1.45-3.16	<0.001
T3	3.00	2.41-3.73	<0.001	8.41	5.44-13.02	<0.001	2.24	1.73-2.89	<0.001	2.49	1.47-4.25	0.001
T4	5.69	4.24-7.65	<0.001	26.08	16.36-41.56	<0.001	3.02	2.04-4.46	<0.001	2.68	1.35-5.34	0.005
N stage												
N0	Reference			Reference			Reference			Reference		
N1	1.32	1.07-1.64	0.011	3.83	2.67-5.50	<0.001	1.17	0.92-1.50	0.208	1.63	1.04-2.54	0.031
N2	1.73	1.04-2.88	0.035	6.93	3.52-13.64	<0.001	2.19	1.27-3.78	0.005	2.67	1.24-5.75	0.012
N3	2.96	1.74-5.02	<0.001	16.3	8.99-29.54	<0.001	0.65	0.34-1.25	0.194	1.23	0.55-2.75	0.612
M stage												
M0	Reference			Reference			Reference			Reference		
M1	8.18	6.2-10.78	< 0.001	35.09	24.54-50.16	<0.001	2.38	1.68-3.38	<0.001	4.27	2.61-6.98	<0.001
Surgery												
No	Reference			Reference			Reference			Reference		
Mastectomy	0.15	0.13-0.19	<0.001	0.06	0.04-0.09	<0.001	0.28	0.23-0.36	<0.001	0.12	0.07~0.19	<0.001
Breast-conserving surgery	0.13	0.11-0.15	<0.001	0.04	0.03-0.05	<0.001	0.36	0.28-0.45	<0.001	0.13	0.08-0.21	<0.001
Radiotherapy												
No/unknown	Reference			Reference			Reference			Reference		
Yes	0.35	0.31-0.41	<0.001	0.44	0.33-0.61	<0.001	0.47	0.40-0.56	<0.001	0.83	0.57-1.21	0.329
Chemotherapy												
No/unknown	Reference			Reference			Reference			Reference		
Yes	0.52	0.41-0.66	<0.001	2.19	1.57-3.07	<0.001	0.71	0.54-0.95	0.021	1.45	0.93-2.26	0.101

OS, overall survival; BCSS, breast cancer-specific survival; HR, hazard ratio; CI, confidence internal.

Multivariate Cox regression analysis further delineated independent prognostic factors. Advanced age, higher T stage, N3 stage, and M1 stage were associated with poorer OS. In contrast, being married and having a household income exceeding $60,000 correlated with improved OS. Additionally, undergoing surgery, radiotherapy, and chemotherapy conferred a survival benefit. For BCSS, advanced age (≥ 66 years), higher tumor grade (II and III), HR−/HER2− subtype, higher T stage, N2–3 stage, and M1 stage were associated with poorer prognosis, whereas marriage, higher household income, and surgical intervention were linked to better BCSS.

### Establishment and evaluation of prognostic models

Significant prognostic features were incorporated into ML models to predict OS and BCSS in patients with MBC at 3-, 5-, and 7-year intervals. **Table 3** presents the predictive performance of ten ML models in both the training and internal test cohorts. Among them, XGBoost demonstrated superior predictive accuracy, achieving AUC values of 0.833 (training) and 0.839 (internal test) for 3-year OS, 0.856 (training) and 0.816 (internal test) for 5-year OS, and 0.843 (training) and 0.830 (internal test) for 7-year OS. Similarly, for BCSS, XGBoost exhibited robust performance with AUC values of 0.944 (training) and 0.872 (internal test) for 3-year BCSS, 0.905 (training) and 0.908 (internal test) for 5-year BCSS, and 0.907 (training) and 0.905 (internal test) for 7-year BCSS. Other machine learning models, such as LR, LightGBM, RF, GNB, CNB, MLP, SVM, and KNN, generally demonstrated slightly lower predictive performance than XGBoost and AdaBoost in the internal test group. For instance, LR exhibited AUC values of 0.828, 0.791, and 0.816 for 3-, 5-, and 7-year OS, respectively, and 0.847, 0.878, and 0.913 for BCSS. LightGBM’s performance was less robust, with AUC values of 0.648, 0.554, and 0.546 for 3-, 5-, and 7-year OS, and 0.763, 0.752, and 0.752 for BCSS. RF showed stronger performance compared to LightGBM, with AUCs of 0.799, 0.773, and 0.777 for OS and 0.862, 0.869, and 0.841 for BCSS. GNB and CNB also exhibited moderate predictive performance, with GNB achieving AUC values of 0.819, 0.793, and 0.811 for OS, and 0.838, 0.865, and 0.812 for BCSS. CNB’s results were similar, with AUCs of 0.792, 0.754, and 0.788 for OS, and 0.818, 0.827, and 0.847 for BCSS. MLP, SVM, and KNN performed less effectively, particularly for 3- and 5-year OS and BCSS predictions, with MLP showing AUCs of 0.583, 0.515, and 0.805 for OS, and 0.515, 0.598, and 0.603 for BCSS. SVM and KNN also displayed suboptimal performance, particularly for 3- and 5-year predictions. In contrast, XGBoost and AdaBoost models excelled, with XGBoost achieving AUC values of 0.847, 0.813, and 0.830 for 3-, 5-, and 7-year OS, and 0.865, 0.870, and 0.903 for BCSS, while AdaBoost followed closely with similarly strong results. Thus, XGBoost and AdaBoost outperformed other models in both OS and BCSS predictions for patients with MBC.

**Table 3:** Performance of machine learning prognostic models in the training and internal test groups.

Groups	Model performance	XGB	LR	LightGBM	RF	AdaBoost	GNB	CNB	MLP	SVM	KNN
Training group	3-year OS	0.833	0.801	0.618	0.908	0.822	0.803	0.779	0.521	0.564	0.787
	5-year OS	0.856	0.819	0.651	0.907	0.838	0.813	0.789	0.502	0.506	0.829
	7-year OS	0.843	0.795	0.666	0.896	0.818	0.791	0.762	0.646	0.568	0.825
	3-year BCSS	0.948	0.873	0.791	0.976	0.916	0.876	0.856	0.584	0.858	0.938
	5-year BCSS	0.905	0.864	0.744	0.976	0.914	0.873	0.805	0.568	0.849	0.912
	7-year BCSS	0.907	0.829	0.684	0.967	0.874	0.883	0.749	0.581	0.821	0.894
Internal test group	3-year OS	0.839	0.828	0.648	0.799	0.847	0.819	0.792	0.583	0.561	0.643
	5-year OS	0.816	0.791	0.554	0.773	0.813	0.793	0.754	0.515	0.608	0.692
	7-year OS	0.830	0.816	0.546	0.777	0.830	0.811	0.788	0.805	0.554	0.774
	3-year BCSS	0.896	0.847	0.763	0.862	0.865	0.838	0.818	0.515	0.600	0.700
	5-year BCSS	0.908	0.878	0.752	0.869	0.870	0.865	0.827	0.598	0.813	0.769
	7-year BCSS	0.905	0.913	0.752	0.841	0.903	0.812	0.847	0.603	0.821	0.820

AUC, area under the curve; XGBoost, extreme gradient boosting; LR, logistic regression; LightGBM, light gradient boosting machine; RF, random forest; AdaBoost, adaptive boosting; GNB, gaussian naive bayes; CNB, complement naive bayes; MLP, multi-layer perceptron neural networks; SVM, support vector machine; KNN, k-nearest neighbors; OS, overall survival; BCSS, breast cancer-specific survival.

**Figure 2:**
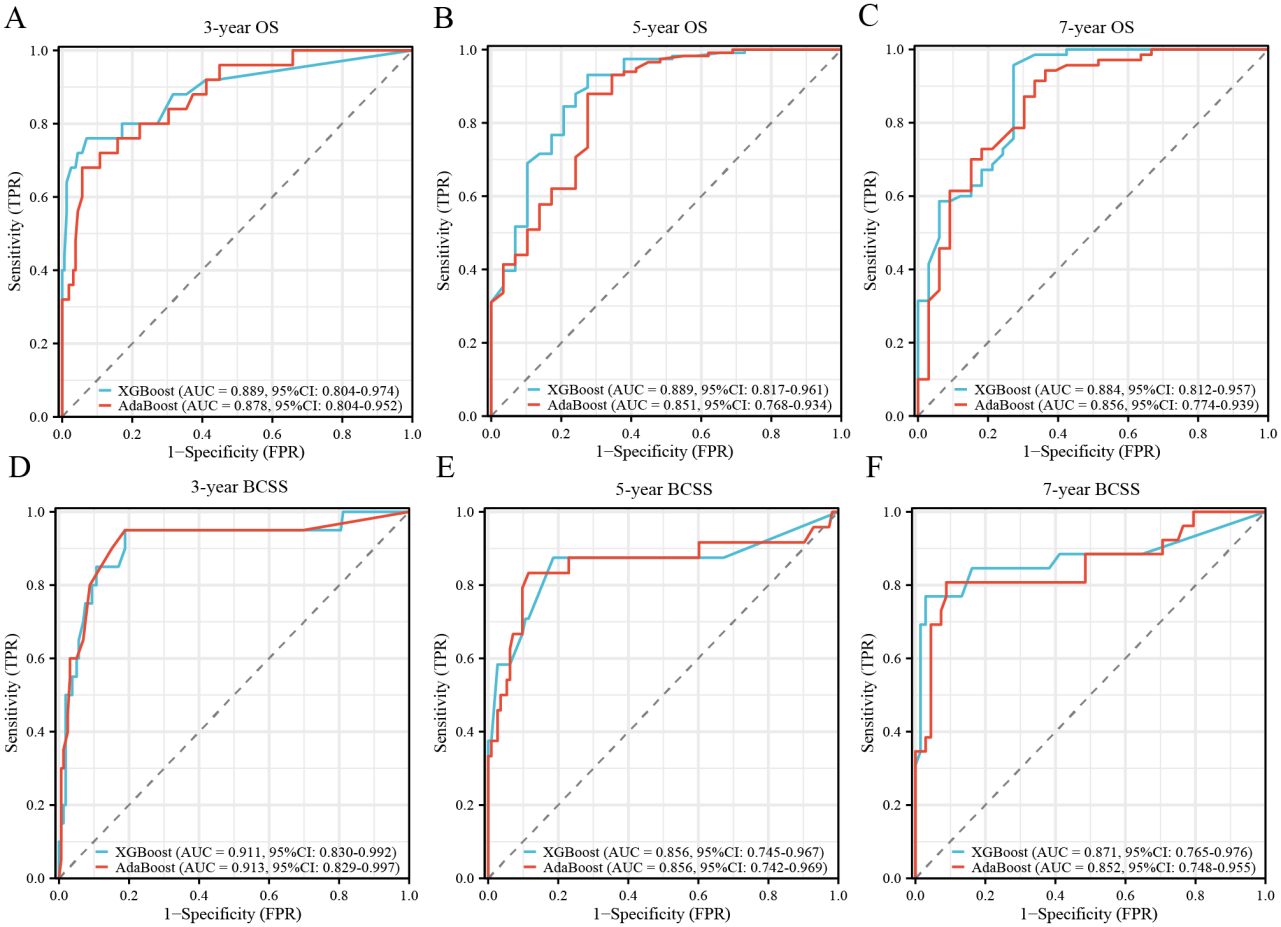
Validation of XGBoost and AdaBoost models from external test group. **(A)** ROC curve for the 3-year OS prognostic model; **(B)** ROC curve for the 5-year OS prognostic model; **(C)** ROC curve for the 7-year OS prognostic model; **(D)** ROC curve for the 3-year BCSS prognostic model; **(E)** ROC curve for the 5-year BCSS prognostic model; **(F)** ROC curve for the 7-year BCSS prognostic model. XGBoost, extreme gradient boosting; AdaBoost, adaptive boosting; ROC, receiver operating characteristic; OS, overall survival; BCSS, breast cancer-specific survival; AUC, area under the curve; CI, confidence internal.

To further validate model robustness and generalizability, an external cohort of 183 patients with MBC from JCH and CHSU was analyzed (**Supplementary Table S1**). In this independent dataset, XGBoost maintained superior predictive performance, with AUC values of 0.889 (3-year OS), 0.889 (5-year OS), and 0.884 (7-year OS) for OS, and 0.911 (3-year BCSS), 0.856 (5-year BCSS), and 0.871 (7-year BCSS) for BCSS. Although AdaBoost also performed well in the external test group, XGBoost remained the optimal model, demonstrating slightly better predictive accuracy (**Figures 2A–F**). Notably, JCH and CHSU cohorts exhibited comparable predictive performance across both models (**Supplementary Figure S2**). Based on these findings, the XGBoost models were identified as the most effective prognostic tools for patients with MBC.

### Evaluation and interpretability of the XGBoost models

**Figure 3:**
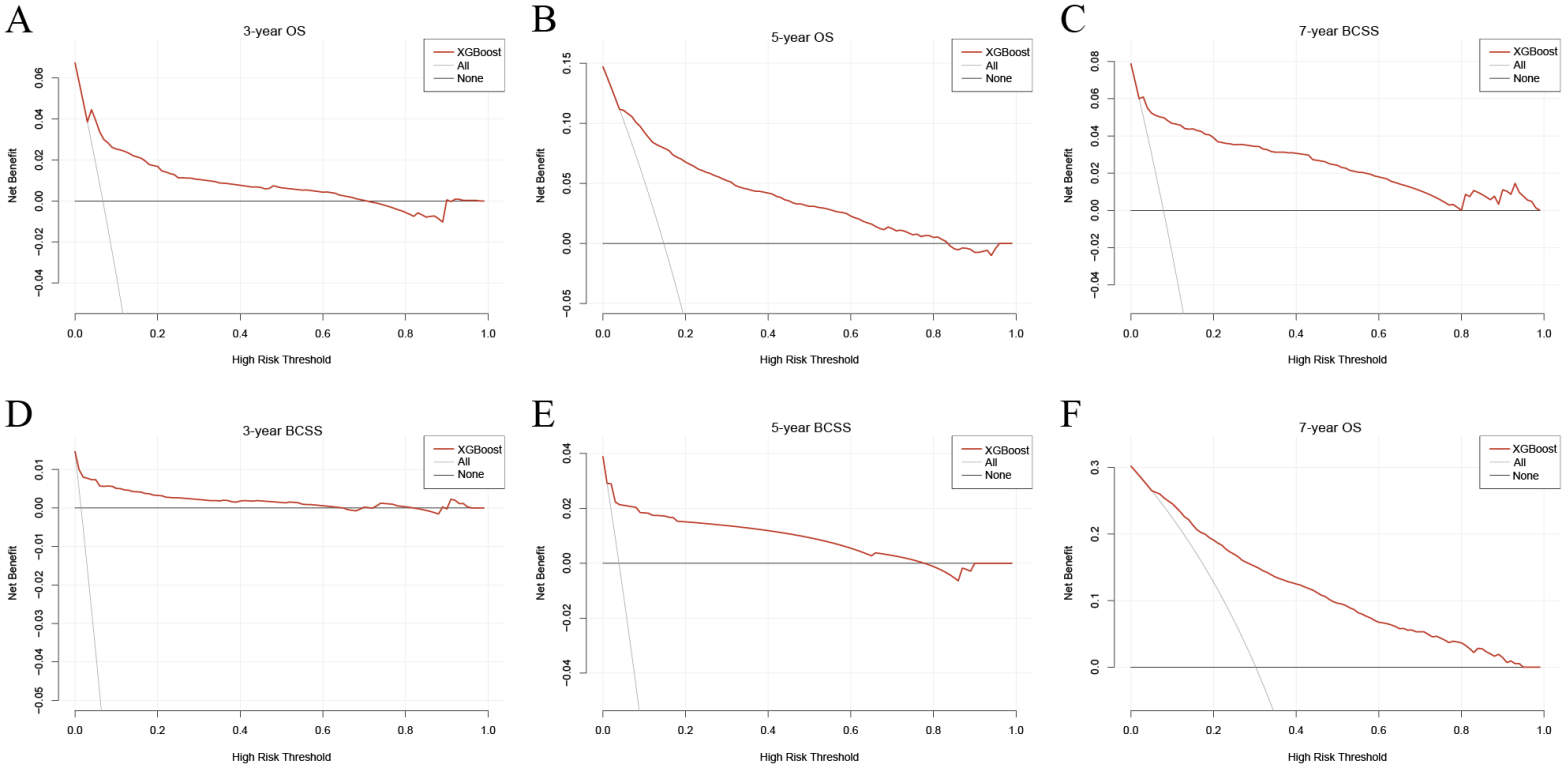
Decision curves for the XGBoost model. **(A)** Decision curve for the 3-year OS prognostic model; **(B)** Decision curve for the 5-year OS prognostic model; **(C)** Decision curve for the 7-year OS prognostic model; **(D)** Decision curve for the 3-year BCSS prognostic model; **(E)** Decision curve for the 5-year BCSS prognostic model; **(F)** Decision curve for the 7-year BCSS prognostic model. XGBoost, extreme gradient boosting; OS, overall survival; BCSS, breast cancer-specific survival.

**Supplementary Table S2** presents the accuracy, sensitivity, specificity, PPV, NPV, and F1 score for all ten ML models. Among them, the XGBoost models demonstrated the highest accuracy, achieving 0.728 for 3-year OS, 0.777 for 5-year OS, and 0.758 for 7-year OS. For BCSS prediction, accuracy values were 0.894 (3-year), 0.887 (5-year), and 0.882 (7-year). The confusion matrix further visualized the classification performance of the XGBoost models in the internal test group (**Supplementary Figure S3**). DCA assessed the clinical applicability of the models, revealing that XGBoost consistently provided a net benefit in survival prediction across all time points, underscoring its clinical utility (**Figure 3**).

**Figure 4:**
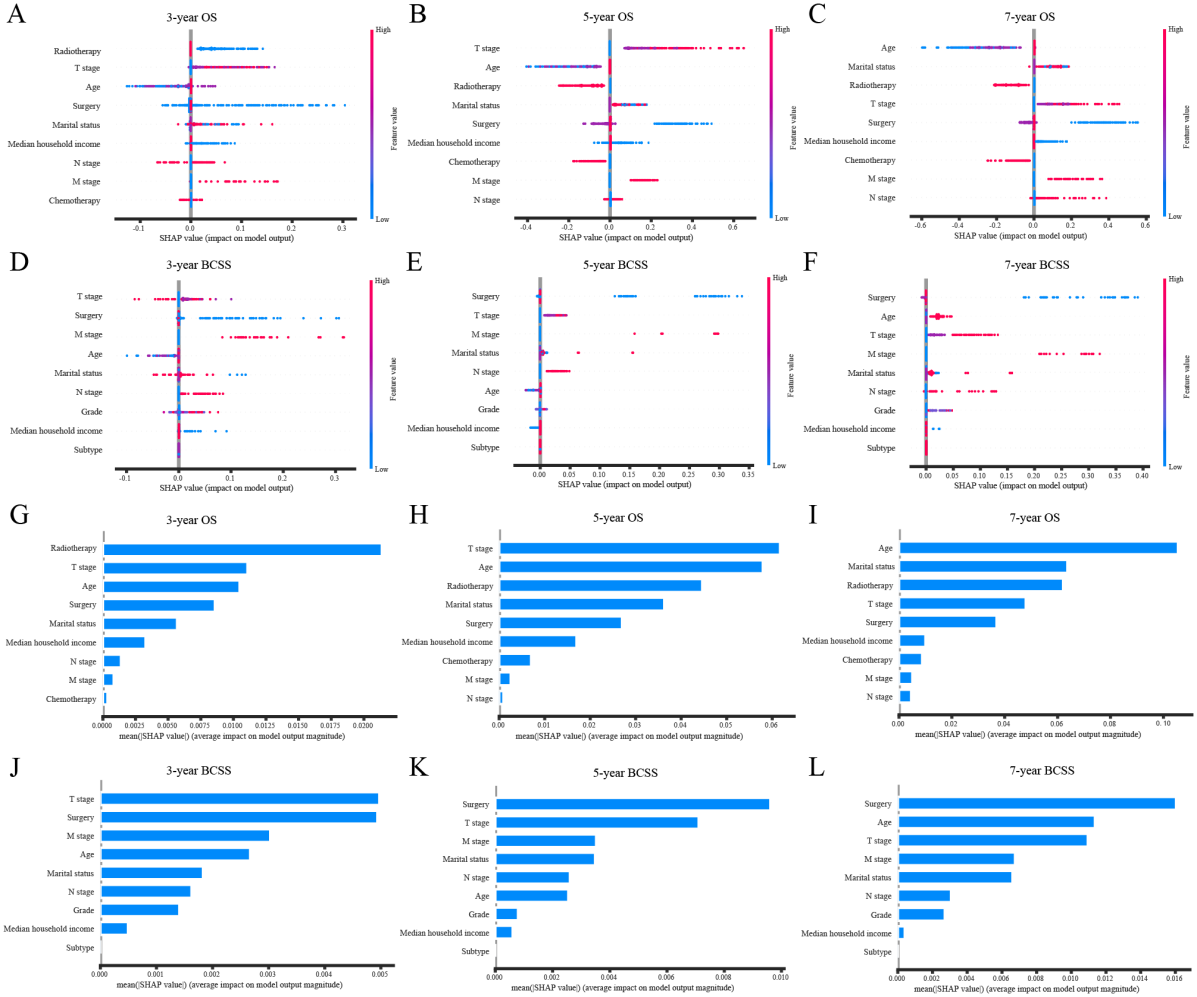
SHAP interprets the XGBoost model. **(A)** SHAP values for each feature at different levels in the 3-year OS prognostic model; **(B)** SHAP values for each feature at different levels in the 5-year OS prognostic model; **(C)** SHAP values for each feature at different levels in the 7-year OS prognostic model; **(D)** SHAP values for each feature at different levels in the 3-year BCSS prognostic model; **(E)** SHAP values for each feature at different levels in the 5-year BCSS prognostic model; **(F)** SHAP values for each feature at different levels in the 7-year BCSS prognostic model; **(G)** Importance of features in the 3-year OS prognostic model; **(H)** Importance of features in the 5-year OS prognostic model; **(I)** Importance of features in the 7-year OS prognostic model; **(J)** Importance of features in the 3-year BCSS prognostic model; **(K)** Importance of features in the 5-year BCSS prognostic model; **(L)** Importance of features in the 7-year BCSS prognostic model. XGBoost, extreme gradient boosting; OS, overall survival; BCSS, breast cancer-specific survival.

SHAP analysis elucidated the contribution of individual features to model predictions. **Figures 4A–F** depict SHAP values for each feature across different levels, with increasing feature values represented in red and decreasing values in blue. Feature importance rankings (**Figures 4G–L**) indicated that radiotherapy, T stage, and age were the most influential predictors of 3-, 5-, and 7-year OS. Similarly, surgery, T stage, and M stage were identified as the key determinants for BCSS prediction.

### Web application development

**Figure 5:**
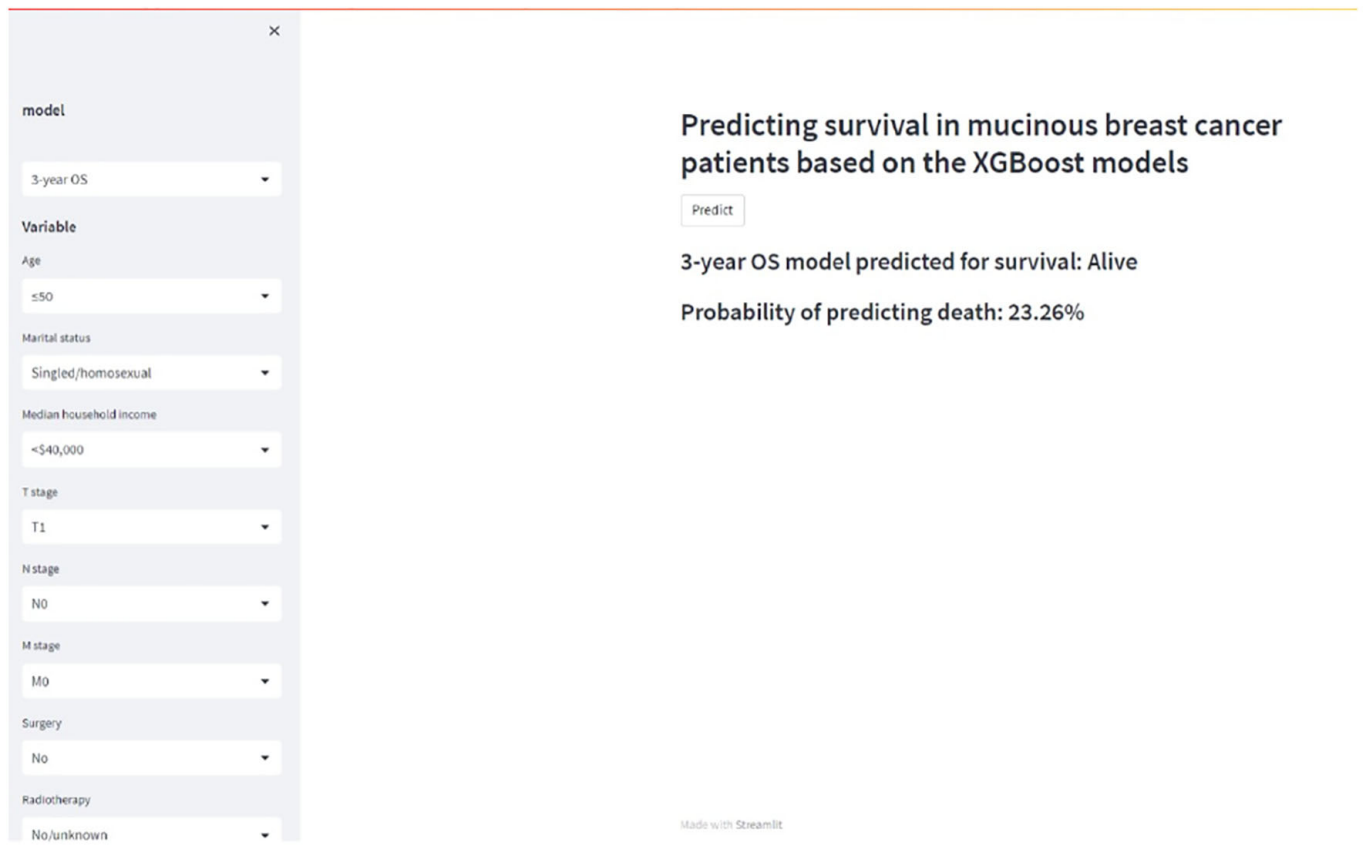
A web calculator for predicting the survival of patients with mucinous breast cancer.

To facilitate widespread adoption of these prognostic models among researchers and clinicians, an interactive web application was developed using the Streamlit platform. This user-friendly tool enables real-time survival probability estimation by inputting clinicopathological parameters (**Figure 5**; https://zqc-mbc-survival.streamlit.app/). By streamlining the integration of predictive models into clinical practice and research, this platform enhances accessibility and usability, providing an efficient resource for MBC prognosis assessment.

### Prognostic impact of surgical approaches in patients with MBC

A total of 4,855 patients with MBC meeting the inclusion criteria were analyzed to assess the impact of mastectomy versus BCS on survival outcomes. Before adjusting for baseline characteristics, both univariate and multivariate Cox regression analyses indicated a significantly improved OS for patients who underwent BCS compared to those who underwent mastectomy. However, no significant difference was observed in BCSS between the two surgical approaches (**Supplementary Table S3**).

**Figure 6:**
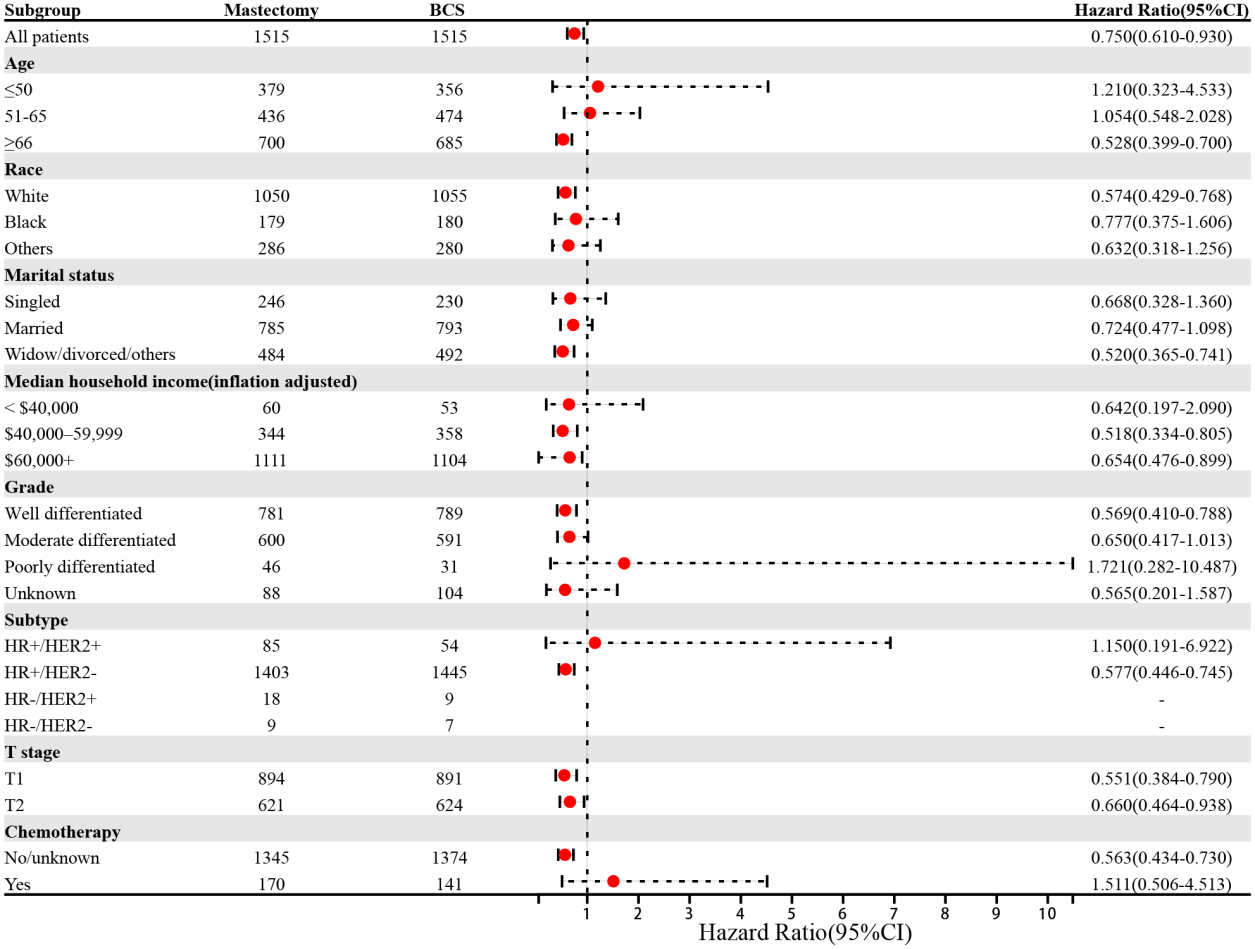
Forest plot of patients with mucinous breast cancer in the subgroup analyses (Mastectomy *vs* BCS). BCS, breast-conserving surgery; CI, confidence internal.

To mitigate baseline imbalances, PSM was applied, yielding a well-balanced cohort with no significant differences in baseline characteristics post-adjustment (**Table 4**). Following PSM adjustment, BCS was associated with a 40% reduction in overall mortality risk compared to mastectomy (**Table 5**, p < 0.001, HR: 0.60, 95% confidence interval [CI]: 0.47–0.77), a finding further substantiated by multivariate Cox regression analyses. However, no significant difference in BC-related mortality was detected between the two groups (p = 0.279, HR: 0.62, 95% CI: 0.26–1.48). To explore variations in OS benefit across different patient subgroups, a forest plot analysis revealed that the survival advantage of BCS was most pronounced among patients aged ≥ 66 years, White individuals, divorced patients, those with a household income >$40,000, grade I tumors, HR+/HER2− subtype, T1 and T2 stage tumors, and those who did not receive chemotherapy (**Figure 6**).

**Table 4:** Comparison of patient characteristics according to surgical approaches before and after propensity score matching.

Variables	Before PSM				After PSM			
	n	Mastectomy	BCS	P	n	Mastectomy	BCS	P
Age								
≤50	840	379 (25.02)	461 (13.80)	<0.001	735	379 (25.02)	356 (23.50)	0.291
51-65	1695	436 (28.78)	1259 (37.69)		910	436 (28.78)	474 (31.29)	
≥66	2320	700 (46.20)	1620 (48.50)		1385	700 (46.20)	685 (45.21)	
Race								
White	3578	1050 (69.31)	2528 (75.69)	<0.001	2105	1050 (69.31)	1055 (69.64)	0.962
Black	550	179 (11.82)	371 (11.11)		359	179 (11.82)	180 (11.88)	
Others	727	286 (18.88)	441 (13.20)		566	286 (18.88)	280 (18.48)	
Marital status								
Singled/homosexual	794	246 (16.24)	548 (16.41)	0.264	476	246 (16.24)	230 (15.18)	0.725
Married	2586	785 (51.82)	1801 (53.92)		1578	785 (51.82)	793 (52.34)	
Widow/divorced/others	1475	484 (31.95)	991 (29.67)		976	484 (31.95)	492 (32.48)	
Median household income (inflation adjusted)								
<$40,000	134	60 (3.96)	74 (2.22)	<0.001	113	60 (3.96)	53 (3.50)	0.692
$40,00-59,999	1049	344 (22.71)	705 (21.11)		702	344 (22.71)	358 (23.63)	
$60,000+	3672	1111 (73.33)	2561 (76.68)		2215	1111 (73.33)	1104 (72.87)	
Grade								
Well differentiated	2732	781 (51.55)	1951 (58.41)	<0.001	1570	781 (51.55)	789 (52.08)	0.225
Moderate differentiated	1765	600 (39.60)	1165 (34.88)		1191	600 (39.60)	591 (39.01)	
Poorly differentiated	107	46 (3.04)	61 (1.83)		77	46 (3.04)	31 (2.05)	
Unknown	251	88 (5.81)	163 (4.88)		192	88 (5.81)	104 (6.86)	
Subtype								
HR+/HER2+	210	85 (5.61)	125 (3.74)	<0.001	139	85 (5.61)	54 (3.56)	0.053
HR+/HER2-	4594	1403 (92.61)	3191 (95.54)		2848	1403 (92.61)	1445 (95.38)	
HR-/HER2+	32	18 (1.19)	14 (0.42)		27	18 (1.19)	9 (0.59)	
HR-/HER2-	19	9 (0.59)	10 (0.30)		16	9 (0.59)	7 (0.46)	
T stage								
T1	3413	894 (59.01)	2519 (75.42)	<0.001	1785	894 (59.01)	891 (58.81)	0.912
T2	1442	621 (40.99)	821 (24.58)		1245	621 (40.99)	624 (41.19)	
Chemotherapy								
Unknown	4437	1345 (88.78)	3092 (92.57)	<0.001	2719	1345 (88.78)	1374 (90.69)	0.083
Yes	418	170 (11.22)	248 (7.43)		311	170 (11.22)	141 (9.31)	

PSM, propensity score matching; BCS, breast-conserving surgery.

**Table 5:** Univariate and multivariate Cox analyses in patients with mucinous breast cancer after propensity score matching.

Variables	Univariate Cox analysis						Multivariate Cox analysis					
	OS			BCSS			OS			BCSS		
	HR	95%CI	P	HR	95%CI	P	HR	95%CI	P	HR	95%CI	P
Age												
≤50	Reference			Reference			Reference			Reference		
51-65	3.25	1.57-6.75	0.002	0.00	0-infinity	0.996	3.09	1.48-6.44	0.003	0.00	0-infinity	0.997
66+	13.58	6.97-26.45	< 0.001	3.58	1.06-12.09	0.04	11.90	5.99-23.64	<0.001	4.61	1.27-16.69	0.02
Race												
White	Reference			Reference			Reference			Reference		
Black	1.00	0.68-1.46	0.997	2.26	0.82-6.29	0.117	/	/	/	/	/	/
Others	0.73	0.51-1.05	0.091	0.89	0.26-3.09	0.852	/	/	/	/	/	/
Marital status												
Singled/homosexual	Reference			Reference			Reference			Reference		
Married	0.77	0.52-1.15	0.201	0.28	0.09-0.86	0.027	0.66	0.44-0.99	0.043	0.22	0.07-0.69	0.01
Widow/divorced/others	1.96	1.34-2.86	< 0.001	0.78	0.28-2.14	0.624	1.06	0.72-1.56	0.774	0.36	0.12-1.05	0.062
Median household income (inflation adjusted)												
<$40,000	Reference			Reference			Reference			Reference		
$40,000-59,999	1.05	0.59-1.89	0.859	0.39	0.08-2.03	0.265	/	/	/	/	/	/
$60,000+	0.68	0.39-1.20	0.188	0.43	0.10-1.87	0.26	/	/	/	/	/	/
Grade												
Well differentiated	Reference			Reference			Reference			Reference		
Moderate differentiated	0.83	0.64-1.08	0.166	2.93	1.17-7.34	0.022	0.90	0.69-1.18	0.446	3.15	1.25-7.95	0.015
Poorly differentiated	0.66	0.27-1.61	0.364	0.00	0-infinity	0.997	1.07	0.41-2.75	0.894	0.00	0-infinity	0.999
Unknown	0.52	0.31-0.88	0.016	1.50	0.31-7.24	0.614	0.59	0.35-1.01	0.052	1.83	0.38-8.87	0.451
Subtype												
HR+/HER2+	Reference			Reference			Reference			Reference		
HR+/HER2-	2.31	0.95-5.59	0.064	3.05E+07	0-infinity	0.998	1.46	0.58-3.71	0.422	/	/	/
HR-/HER2+	0.88	0.10-7.54	0.908	1.00	0-infinity	1	0.67	0.08-6.05	0.724	/	/	/
HR-/HER2-	6.74	1.61-28.22	0.009	3.77E+08	0-infinity	0.997	2.60	0.61-11.09	0.197	/	/	/
T stage												
T1	Reference			Reference			Reference			Reference		
T2	1.65	1.30-2.11	< 0.001	2.41	1.03-5.64	0.043	1.58	1.24-2.02	<0.001	2.24	0.95-5.25	0.064
Surgery												
Mastectomy		Reference			Reference			Reference			Reference	
Breast-conserving surgery	0.60	0.47-0.77	< 0.001	0.60	0.25-1.43	0.249	0.60	0.47-0.78	<0.001	0.62	0.26-1.48	0.279
Chemotherapy												
No/unknown	Reference			Reference			Reference			Reference		
Yes	0.45	0.26-0.79	0.005	1.94	0.66-5.73	0.231	1.09	0.58-2.08	0.786	/	/	/

OS, overall survival; BCSS, breast cancer-specific survival; HR, hazard ratio; CI, confidence internal.

## Discussion

MBC, as a rare histological subtype, has received limited attention due to its relatively favorable prognosis (26, 27). The majority of MBC cases belong to the ER+/HER2− molecular subtype, and treatment strategies typically align with those established for IDC, emphasizing surgery, chemotherapy, and endocrine therapy (28). However, genomic landscape analysis by Pareja et al. has demonstrated that MBC exhibits distinct genetic heterogeneity compared to other common ER+/HER2− breast cancers (7), underscoring the necessity for personalized treatment approaches and tailored prognostic models. Previous prognostic models for MBC have shown limitations. Gao et al. developed a nomogram for MBC prognosis prediction, but its predictive performance was suboptimal (C-index = 0.680) (13). Fu and Zhu et al. constructed nomograms for OS and BCSS with improved C-indices (0.803–0.816) but lacked external validation (14, 15). To our knowledge, this study represents the largest comprehensive analysis of MBC prognosis and surgical approaches to date. It is also the first to develop OS and BCSS prediction models using ten ML algorithms, with XGBoost demonstrating superior sensitivity, specificity, and accuracy across 3-, 5-, and 7-year survival predictions. Furthermore, this study is the first to apply PSM in evaluating the survival benefits of mastectomy versus BCS in patients with MBC, providing robust evidence to guide surgical decision-making.

Several independent risk factors significantly associated with both OS and BCSS were identified, including age ≥ 66 years, higher T stage, N2 stage, and M1 stage. Conversely, protective factors included being married, a household income exceeding $60,000, and undergoing surgery. Recent studies have demonstrated that advanced age is linked to poorer OS and BCSS, with reported age cut-offs of 52, 65, and 80 years (13, 15, 29). Consistent with established oncologic principles, higher TNM stage was confirmed as a negative prognostic indicator in MBC. Marital status has been widely recognized as a significant predictor of survival in patients with BC (30–34), with married individuals exhibiting better quality of life and improved survival compared to unmarried or divorced counterparts (35). Moreover, higher-income households are more likely to adhere to medical recommendations, benefiting from optimized therapeutic decision-making without financial constraints (36, 37). In line with this, our findings revealed that patients with a family income above $60,000 had superior prognoses. Extensive research has established that surgical intervention, whether mastectomy or BCS, improves survival outcomes by reducing the primary tumor burden (38–41), aligning with our results. Additionally, radiotherapy and chemotherapy were identified as independent prognostic factors for OS but not BCSS. Mo et al. previously reported radiotherapy as a determinant of BCSS in MBC individuals with T1–2N0M0 tumors (T ≤ 3 cm) (42), suggesting that its survival benefit may be restricted to specific subgroups. However, in our analysis of the overall MBC population, no significant association with BCSS was observed. Similarly, prior studies have indicated that chemotherapy enhances OS after PSM, but this benefit does not extend to BCSS (43), a finding corroborated by our results.

Based on performance metrics, XGBoost and AdaBoost were selected from the training and internal test groups for further evaluation. When external test data were applied, XGBoost consistently outperformed AdaBoost, confirming its superiority in predictive accuracy. Among the ten ML models compared, XGBoost emerged as the best-performing algorithm. Both XGBoost and AdaBoost, as ensemble learning methods, are particularly effective in handling complex nonlinear relationships (44, 45). However, XGBoost incorporates a regularization mechanism that mitigates overfitting and enhances generalization, a critical advantage when working with high-dimensional medical data and relatively small sample sizes. Previous prognostic models for MBC have demonstrated limited predictive accuracy. Fu et al. developed a nomogram for 5- and 7-year BCSS in patients with early-stage MBC, achieving a C-index of 0.789 (14). In contrast, our XGBoost model exhibited superior predictive power, with AUC values of 0.905 and 0.907 for 5- and 7-year BCSS in the training group. When externally validated, the model maintained its robustness, achieving AUCs of 0.856 and 0.871, respectively. Similarly, Zhu et al. proposed a prognostic nomogram for 3- and 5-year OS in patients with MBC, reporting a C-index of 0.803 (15), while Gao et al. developed a nomogram for 5- and 10-year OS with AUC values of 0.714, 0.813, and 0.805 across training, internal validation, and external validation cohorts, respectively (13). In comparison, our XGBoost models demonstrated superior predictive performance, with AUC values of 0.833, 0.839, and 0.889 for 3-year OS across the training, internal test, and external validation cohorts, and AUC values of 0.856, 0.816, and 0.889 for 5-year OS in the respective groups. These results highlight the significantly enhanced prognostic accuracy of our XGBoost models compared to prior nomograms, providing a more reliable framework for clinical decision-making and patient stratification. The interpretability of our XGBoost models were enhanced using SHAP analysis, which identified radiotherapy, T stage, age, surgery, and M stage as key predictors of prognosis. Specifically, receiving radiotherapy, presenting with a lower T stage, younger age, undergoing surgery, and an M0 stage were associated with improved prognosis and higher survival probabilities. Furthermore, DCA confirmed the exceptional clinical utility of our XGBoost model. To facilitate clinical implementation, an interactive web-based tool has been developed, enabling clinicians to rapidly estimate individualized survival probabilities for patients with MBC.

Since the landmark NSABP B-06 trial, it has been well established that patients with early-stage BC undergoing BCS achieve survival outcomes comparable to those undergoing mastectomy (46). Subsequent large-scale studies further demonstrated superior survival in patients with early-stage BC treated with BCS combined with radiotherapy compared to those who underwent mastectomy without radiotherapy (47, 48). As a result, clinicians increasingly favor BCS with radiotherapy over mastectomy for eligible patients. However, the survival advantage of BCS with radiotherapy versus mastectomy in patients with MBC remains unconfirmed. To address this, our study focused on MBC individuals with stage T1–2N0M0 and applied PSM to mitigate confounding effects, thereby approximating a randomized comparison of survival benefits between the BCS and mastectomy groups. After PSM, OS in the BCS group was significantly higher than in the mastectomy group (p < 0.001, HR = 0.60, 95% CI: 0.47–0.78). However, no significant difference was observed in BCSS between the two groups (p = 0.279, HR = 0.62, 95% CI: 0.26–1.48). These results align with those reported by Yu et al. (24), despite their study lacking PSM adjustment for potential confounding biases. Thus, our study provides strong evidence that MBC individuals with stage T1–2N0M0 may benefit from BCS with radiotherapy in terms of improved OS.

Despite these strengths, several limitations must be acknowledged. First, as a retrospective study, selection bias and unmeasured confounding factors cannot be entirely excluded, necessitating validation in a prospective cohort. Second, the SEER database lacks information on endocrine and targeted therapies, both of which significantly influence prognosis, potentially limiting model performance. Third, the absence of endocrine therapy data led to the exclusion of older patients with stage T1 disease who underwent BCS and received endocrine therapy without radiotherapy, introducing a potential selection bias in the survival comparison between mastectomy and BCS. Finally, considering that the median follow-up time in the SEER database is only five years, the reliability of our model in predicting long-term survival may be limited.

## Conclusion

In conclusion, we developed six optimized prognostic models using the XGBoost algorithm to predict survival in patients with MBC, with external validation confirming their high generalizability. Notably, our findings demonstrated a significant OS benefit for patients undergoing BCS.

## Data Availability

The raw data supporting the conclusions of this article will be made available by the authors, without undue reservation.

## Glossary

AdaBoost: adaptive boosting
AUC: area under the curve
BC: breast cancer
BCS: breast-conserving surgery
BCSS: breast cancer-specific survival
CHSU: Cancer Hospital of Shantou University Medical College
CI: confidence internal
C-index: concordance index
CNB: complement naive bayes
DCA: decision curve analysis
GNB: gaussian naive bayes
IDC: infiltrating ductal carcinoma
JCH: Jiangmen Central Hospital
KNN: k-nearest neighbors
LightGBM: light gradient boosting machine
LR: logistic regression
MBC: mucinous breast cancer
ML: machine learning
MLP: multi-layer perceptron neural networks
NPV: negative predictive value
OS: overall survival
PPV: positive predictive value
PSM: propensity score matching
RF: random forest
SEER: Surveillance Epidemiology and End Results
SHAP: SHapley Additive exPlanations
SVM: support vector machine
XGBoost: extreme gradient boosting
