# Supplementary material for: Development and validation of novel machine learning-based prognostic models and propensity score matching for comparison of surgical approaches in mucinous breast cancer
Source: Front Endocrinol (Lausanne). 2025 Jun 3;16:1557858. doi: 10.3389/fendo.2025.1557858 (PMC12170503; doi:10.3389/fendo.2025.1557858)
Supplement: Supplementary file 1 [file DataSheet1.docx]

**Development and validation of novel machine learning-based prognostic models and propensity score matching for comparison of surgical approaches in mucinous breast cancer**


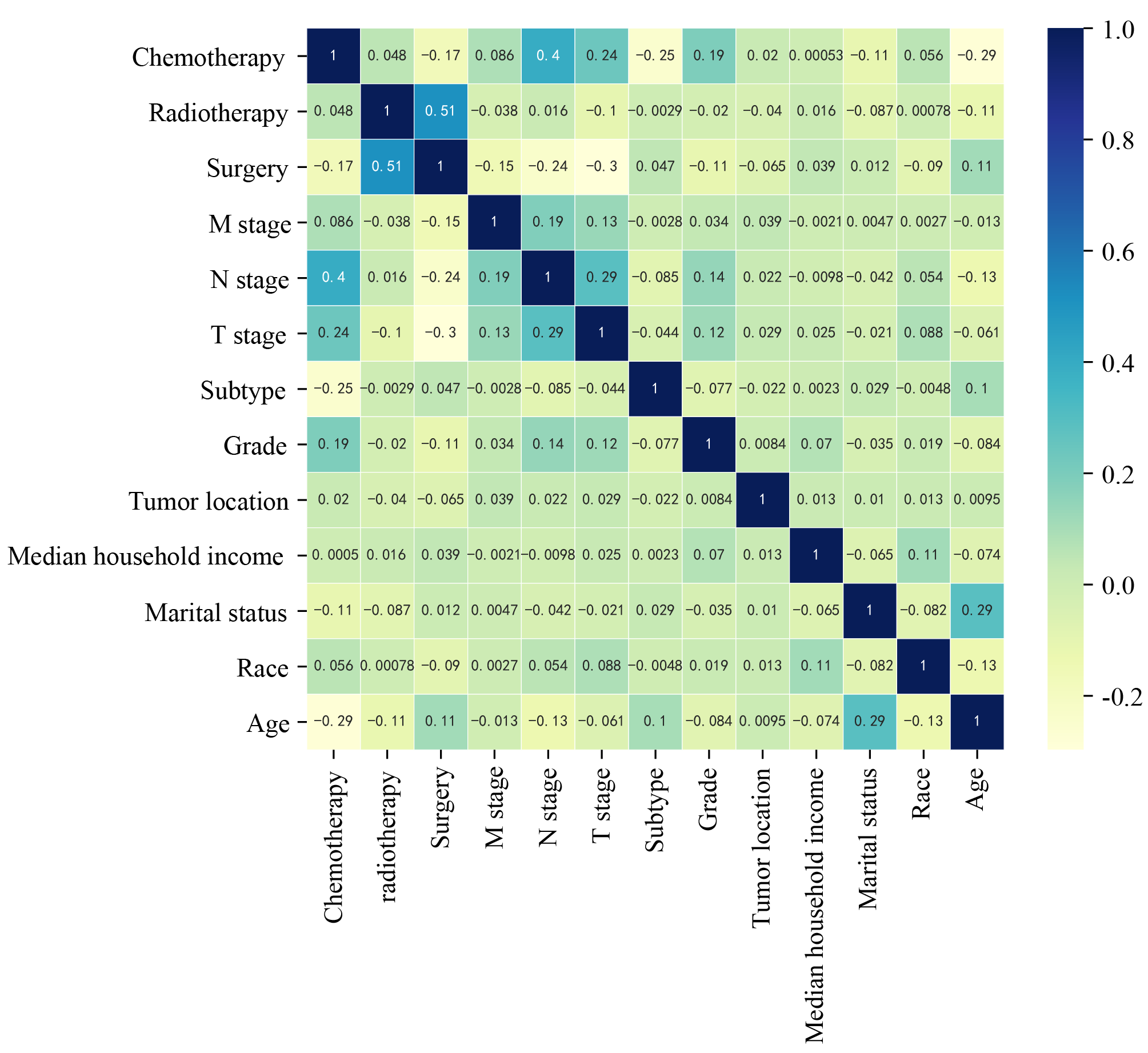


Supplementary Figure.1 Correlation between clinical characteristic data.


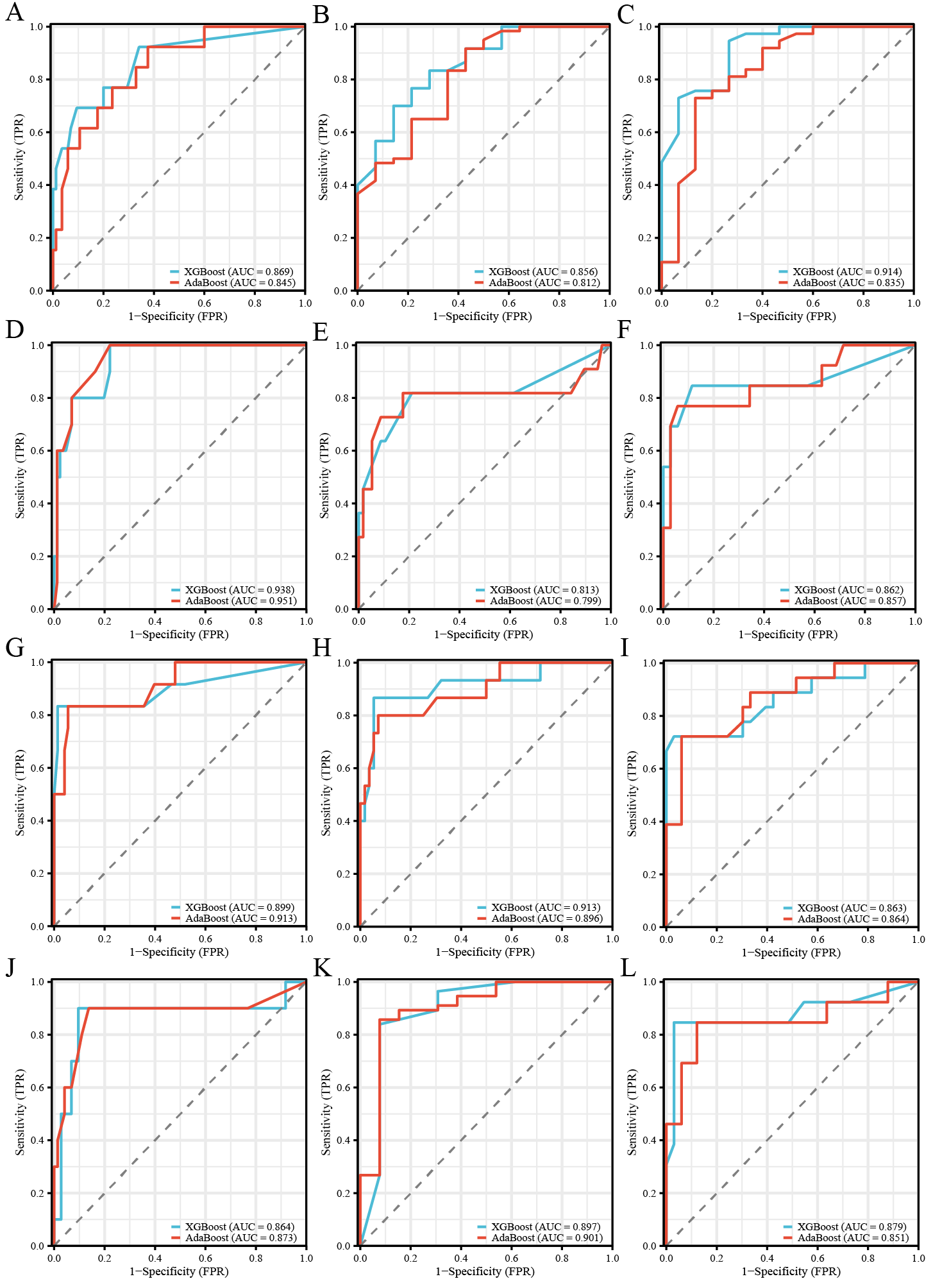


Supplementary Figure.2 Validation of XGBoost and AdaBoost models from external test group. **A** ROC curve for the 3-year OS prognostic model from the JCH cohort; **B** ROC curve for the 5-year OS prognostic model the JCH cohort; **C** ROC curve for the 7-year OS prognostic model the JCH cohort; **D** ROC curve for the 3-year BCSS prognostic model the JCH cohort; **E** ROC curve for the 5-year BCSS prognostic model the JCH cohort; **F** ROC curve for the 7-year BCSS prognostic model the JCH cohort. **G** ROC curve for the 3-year OS prognostic model from the CHSU cohort; **H** ROC curve for the 5-year OS prognostic model the CHSU cohort; **I** ROC curve for the 7-year OS prognostic model the CHSU cohort; **J** ROC curve for the 3-year BCSS prognostic model the CHSU cohort; **K** ROC curve for the 5-year BCSS prognostic model the CHSU cohort; **L** ROC curve for the 7-year BCSS prognostic model the CHSU cohort. XGBoost, extreme gradient boosting; AdaBoost, adaptive boosting; ROC, receiver operating characteristic; OS, overall survival; BCSS, breast cancer-specific survival; JCH, XX Hospital; CHSU, Cancer Hospital of XXX; AUC, area under the curve; CI, confidence internal.


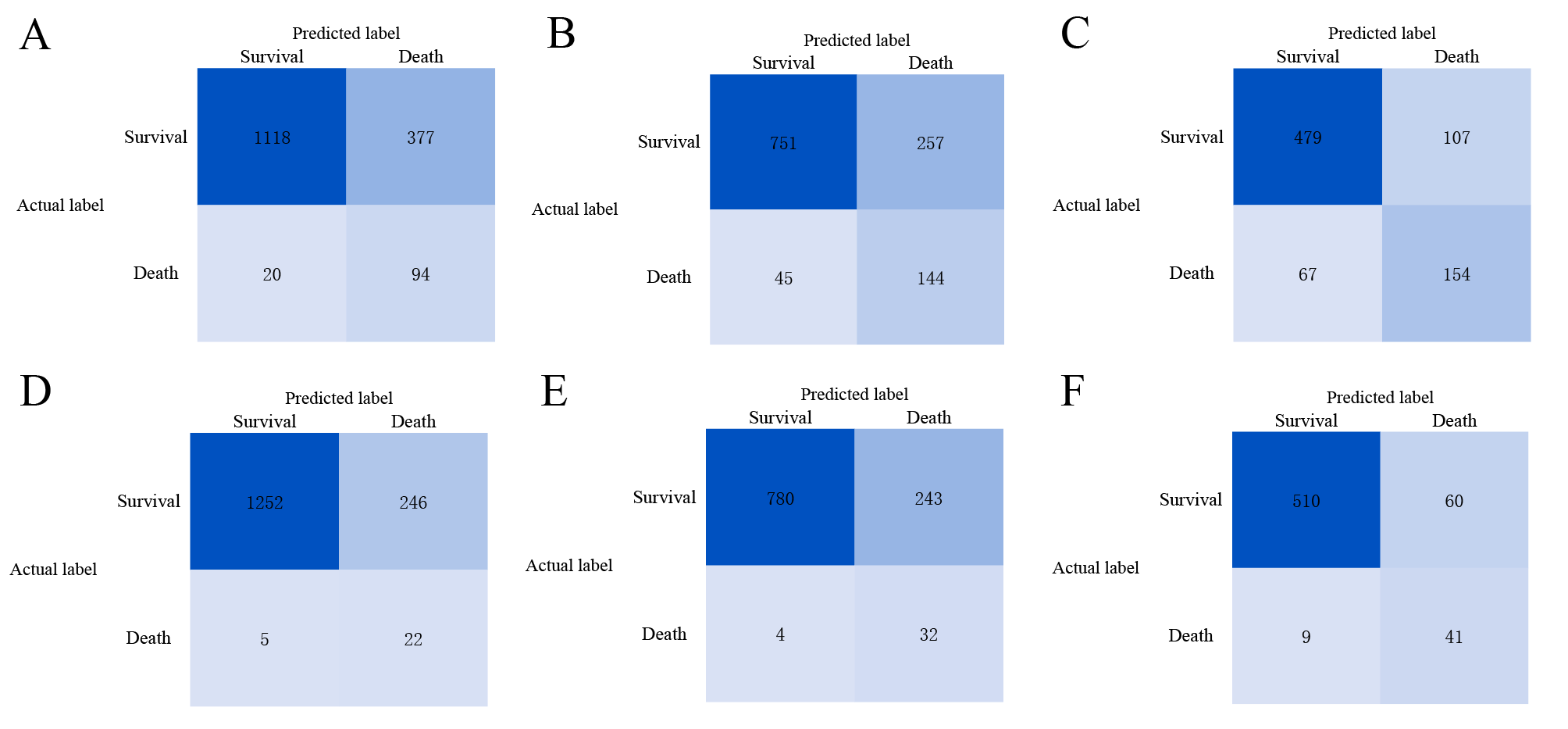


Supplementary Figure.3 Confusion matrix of the XGBoost model's predicted results in the internal test group. **A** Confusion matrix of the 3-year OS prognostic model; **B** Confusion matrix of the 5-year OS prognostic model; **C** Confusion matrix of the 7-year OS prognostic model; **D** Confusion matrix of the 3-year BCSS prognostic model; **E** Confusion matrix of the 5-year BCSS prognostic model; **F** Confusion matrix of the 7-year BCSS prognostic model. XGBoost, extreme gradient boosting; OS, overall survival; BCSS, breast cancer-specific survival.

Table S1. Baseline characteristics of patients with mucinous breast cancer in the external test group

| **Variables** |  | **n, %** | |
| --- | --- | --- | --- |
|  |  | **JCH** | **CHSU** |
| Age | ≤50 | 48 (49.0) | 27 (31.8) |
|  | 51-65 | 24 (24.5) | 21 (24.7) |
|  | ≥66 | 26 (26.5) | 37 (43.5) |
| Race | White | 0 (0.0) | 0 (0.0) |
|  | Black | 0 (0.0) | 0 (0.0) |
|  | Others | 98 (100.0) | 85 (100.0) |
| Marital status | Singled/homosexual | 7 (7.1) | 10 (11.8) |
|  | Married | 88 (89.8) | 52 (61.2) |
|  | Widow/divorced/others | 3 (3.1) | 23 (27.1) |
| Median household income (inflation adjusted) | <$40,000 | 10 (10.2) | 3 (3.5) |
|  | $40,00-59,999 | 38 (38.8) | 26 (30.6) |
|  | $60,000+ | 50 (51.0) | 56 (65.9) |
| Grade | Well differentiated | 21 (21.4) | 44 (51.8) |
|  | Moderate differentiated | 76 (77.6) | 36 (42.4) |
|  | Poorly differentiated | 1 (1.0) | 4 (4.7) |
|  | Unkown | 0 (0.0) | 1 (1.2) |
| Subtype | HR+/HER2+ | 14 (14.3) | 7 (8.2) |
|  | HR+/HER2- | 82 (83.7) | 76 (89.4) |
|  | HR-/HER2+ | 2 (2.0) | 0 (0.0) |
|  | HR-/HER2- | 0 (0.0) | 2 (2.4) |
| T stage | T1 | 47 (48.0) | 44 (51.8) |
|  | T2 | 45 (45.9) | 25 (29.4) |
|  | T3 | 4 (4.1) | 11 (12.9) |
|  | T4 | 2 (2.0) | 5 (5.9) |
| N stage | N0 | 69 (70.4) | 65 (76.5) |
|  | N1 | 17 (17.3) | 16 (18.8) |
|  | N2 | 6 (6.1) | 1 (1.2) |
|  | N3 | 6 (6.1) | 3 (3.5) |
| M stage | M0 | 91 (92.9) | 82 (96.5) |
|  | M1 | 7 (7.1) | 3 (3.5) |
| Surgery | No | 9 (9.2) | 7 (8.2) |
|  | Mastectomy | 76 (77.5) | 40 (47.1) |
|  | Breast-conserving surgery | 13 (13.3) | 38 (44.7) |
| Radiotherapy | No/unknown | 75 (76.5) | 45 (52.9) |
|  | Yes | 23 (23.5) | 40 (47.1) |
| Chemotherapy | No/unknown | 71 (72.4) | 67 (78.8) |
|  | Yes | 27 (27.6) | 18 (21.2) |

Abbreviations: JCH, Jiangmen Hospital; CHSU, Cancer Hospital of Shantou University Medical College.

Table S2. Performance of machine learning prognostic models in the training group

| Survival | Indicators | XGBoost | LR | LightGBM | RF | AdaBoost | GNB | CNB | MLP | SVM | KNN |
| --- | --- | --- | --- | --- | --- | --- | --- | --- | --- | --- | --- |
| 3-year OS | Accuracy | 0.728 | 0.749 | 0.933 | 0.787 | 0.733 | 0.709 | 0.767 | 0.654 | 0.631 | 0.927 |
|  | Sensitivity | 0.812 | 0.748 | 0.265 | 0.859 | 0.778 | 0.780 | 0.657 | 0.401 | 0.541 | 0.689 |
|  | Specificity | 0.713 | 0.727 | 0.970 | 0.767 | 0.722 | 0.703 | 0.771 | 0.669 | 0.634 | 0.855 |
|  | PPV | 0.175 | 0.174 | 0.165 | 0.220 | 0.170 | 0.159 | 0.172 | 0.089 | 0.103 | 0.457 |
|  | NPV | 0.980 | 0.973 | 0.933 | 0.986 | 0.977 | 0.977 | 0.968 | 0.941 | 0.950 | 0.953 |
|  | F1‐score | 0.287 | 0.281 | 0.186 | 0.350 | 0.278 | 0.265 | 0.273 | 0.145 | 0.166 | 0.547 |
| 5-year OS | Accuracy | 0.777 | 0.766 | 0.796 | 0.827 | 0.746 | 0.747 | 0.735 | 0.543 | 0.498 | 0.856 |
|  | Sensitivity | 0.770 | 0.722 | 0.527 | 0.812 | 0.785 | 0.736 | 0.699 | 0.559 | 0.643 | 0.780 |
|  | Specificity | 0.777 | 0.767 | 0.804 | 0.818 | 0.738 | 0.747 | 0.739 | 0.539 | 0.473 | 0.753 |
|  | PPV | 0.380 | 0.355 | 0.322 | 0.452 | 0.342 | 0.335 | 0.319 | 0.188 | 0.203 | 0.525 |
|  | NPV | 0.951 | 0.938 | 0.872 | 0.959 | 0.951 | 0.940 | 0.933 | 0.844 | 0.827 | 0.924 |
|  | F1‐score | 0.506 | 0.475 | 0.371 | 0.580 | 0.476 | 0.460 | 0.437 | 0.244 | 0.265 | 0.618 |
| 7-year OS | Accuracy | 0.758 | 0.716 | 0.679 | 0.797 | 0.736 | 0.732 | 0.697 | 0.663 | 0.589 | 0.778 |
|  | Sensitivity | 0.757 | 0.753 | 0.689 | 0.819 | 0.753 | 0.715 | 0.722 | 0.526 | 0.528 | 0.772 |
|  | Specificity | 0.757 | 0.702 | 0.673 | 0.782 | 0.729 | 0.740 | 0.681 | 0.727 | 0.616 | 0.726 |
|  | PPV | 0.580 | 0.523 | 0.507 | 0.627 | 0.546 | 0.545 | 0.499 | 0.447 | 0.398 | 0.651 |
|  | NPV | 0.875 | 0.867 | 0.801 | 0.905 | 0.872 | 0.852 | 0.829 | 0.782 | 0.759 | 0.835 |
|  | F1‐score | 0.655 | 0.616 | 0.552 | 0.710 | 0.633 | 0.618 | 0.590 | 0.468 | 0.427 | 0.701 |
| 3-year BCSS | Accuracy | 0.888 | 0.841 | 0.985 | 0.937 | 0.868 | 0.893 | 0.874 | 0.778 | 0.866 | 0.986 |
|  | Sensitivity | 0.887 | 0.780 | 0.615 | 0.877 | 0.876 | 0.754 | 0.746 | 0.346 | 0.692 | 0.906 |
|  | Specificity | 0.886 | 0.840 | 0.967 | 0.932 | 0.830 | 0.895 | 0.875 | 0.785 | 0.867 | 0.953 |
|  | PPV | 0.106 | 0.072 | 0.278 | 0.374 | 0.089 | 0.096 | 0.082 | 0.132 | 0.246 | 0.537 |
|  | NPV | 0.998 | 0.996 | 0.985 | 0.994 | 0.997 | 0.996 | 0.995 | 0.956 | 0.994 | 0.992 |
|  | F1‐score | 0.189 | 0.131 | 0.304 | 0.357 | 0.161 | 0.169 | 0.148 | 0.175 | 0.278 | 0.670 |
| 5-year BCSS | Accuracy | 0.887 | 0.859 | 0.962 | 0.938 | 0.878 | 0.856 | 0.891 | 0.911 | 0.931 | 0.967 |
|  | Sensitivity | 0.800 | 0.725 | 0.508 | 0.873 | 0.801 | 0.748 | 0.624 | 0.207 | 0.660 | 0.888 |
|  | Specificity | 0.852 | 0.859 | 0.980 | 0.935 | 0.880 | 0.860 | 0.903 | 0.940 | 0.942 | 0.873 |
|  | PPV | 0.232 | 0.185 | 0.355 | 0.379 | 0.214 | 0.180 | 0.207 | 0.165 | 0.390 | 0.572 |
|  | NPV | 0.988 | 0.986 | 0.962 | 0.994 | 0.990 | 0.988 | 0.983 | 0.967 | 0.985 | 0.983 |
|  | F1‐score | 0.355 | 0.291 | 0.396 | 0.527 | 0.337 | 0.288 | 0.308 | 0.225 | 0.465 | 0.695 |
| 7-year BCSS | Accuracy | 0.882 | 0.835 | 0.921 | 0.931 | 0.843 | 0.851 | 0.823 | 0.803 | 0.899 | 0.922 |
|  | Sensitivity | 0.765 | 0.639 | 0.386 | 0.849 | 0.746 | 0.641 | 0.631 | 0.270 | 0.621 | 0.887 |
|  | Specificity | 0.887 | 0.852 | 0.982 | 0.923 | 0.845 | 0.870 | 0.839 | 0.850 | 0.924 | 0.826 |
|  | PPV | 0.404 | 0.286 | 0.139 | 0.576 | 0.317 | 0.300 | 0.255 | 0.148 | 0.425 | 0.529 |
|  | NPV | 0.977 | 0.964 | 0.921 | 0.984 | 0.974 | 0.965 | 0.963 | 0.888 | 0.965 | 0.966 |
|  | F1‐score | 0.518 | 0.386 | 0.278 | 0.675 | 0.437 | 0.406 | 0.361 | 0.286 | 0.498 | 0.657 |

Abbreviations: XGBoost, extreme gradient boosting; LR, logistic regression; LightGBM, light gradient boosting machine; RF, random forest; AdaBoost, adaptive boosting; GNB, gaussian naive bayes; CNB, complement naive bayes; MLP, multi-layer perceptron neural networks; SVM, support vector machine; KNN, k-nearest neighbors; OS, overall survival; BCSS, breast cancer-specific survival; PPV, positive predictive value; NPV, negative predictive value.

Table S3. Univariate and multivariate Cox analyses in patients with mucinous breast cancer before propensity score matching

|  | **Univariate COX analysis** | | | | | | |  | **Multivariate COX analysis** | | | | | | |
| --- | --- | --- | --- | --- | --- | --- | --- | --- | --- | --- | --- | --- | --- | --- | --- |
|  | **OS** | | |  | **BCSS** | | |  | **OS** | | |  | **BCSS** | | |
|  | **HR** | **95%CI** | **P** |  | **HR** | **95%CI** | **P** |  | **HR** | **95%CI** | **P** |  | **HR** | **95%CI** | **P** |
| Age |  | | |  |  | | |  | | | | | | | |
| ≤50 | Reference | | |  | Reference | | |  | Reference | | |  | Reference | | |
| 51-65 | 2.26 | 1.21-4.23 | 0.011 |  | 0.51 | 0.10-2.54 | 0.413 |  | 2.39 | 1.27-4.49 | 0.007 |  | 0.59 | 0.12-2.96 | 0.523 |
| 66+ | 9.86 | 5.53-17.55 | < 0.001 |  | 3.90 | 1.19-12.81 | 0.025 |  | 9.27 | 5.14-16.75 | <0.001 |  | 4.71 | 1.38-16.15 | 0.014 |
| Race |  |  |  |  |  |  |  |  |  |  |  |  |  |  |  |
| White | Reference | | |  | Reference | | |  | Reference | | |  | Reference | | |
| Black | 0.88 | 0.62-1.24 | 0.459 |  | 1.61 | 0.66-3.92 | 0.291 |  | / | / | / |  | / | / | / |
| Others | 0.73 | 0.52-1.01 | 0.061 |  | 0.60 | 0.18-1.99 | 0.404 |  | / | / | / |  | / | / | / |
| Marital status |  |  |  |  |  |  |  |  |  |  |  |  |  |  |  |
| Singled/homosexual | Reference | | |  | Reference | | |  | Reference | | |  | Reference | | |
| Married | 0.89 | 0.64-1.25 | 0.500 |  | 0.40 | 0.16-1.00 | 0.049 |  | 0.69 | 0.49-0.97 | 0.034 |  | 0.30 | 0.12-0.76 | 0.011 |
| Widow/divorced/others | 2.10 | 1.52-2.91 | < 0.001 |  | 1.07 | 0.46-2.50 | 0.874 |  | 1.11 | 0.79-1.55 | 0.560 |  | 0.55 | 0.23-1.33 | 0.186 |
| Median household income (inflation adjusted) |  |  |  |  |  |  |  |  |  |  |  |  |  |  |  |
| <$40,000 | Reference | | |  | Reference | | |  | Reference | | |  | Reference | | |
| $40,000-59,999 | 0.96 | 0.56-1.65 | 0.885 |  | 0.49 | 0.10-2.30 | 0.366 |  | / | / | / |  | / | / | / |
| $60,000+ | 0.60 | 0.35-1.01 | 0.054 |  | 0.50 | 0.12-2.09 | 0.340 |  | / | / | / |  | / | / | / |
| Grade |  |  |  |  |  |  |  |  |  |  |  |  |  |  |  |
| Well differentiated | Reference | | |  | Reference | | |  | Reference | | |  | Reference | | |
| Moderate differentiated | 0.80 | 0.63-1.01 | 0.061 |  | 2.17 | 1.09-4.30 | 0.027 |  | / | / | / |  | 2.24 | 1.12-4.46 | 0.022 |
| Poorly differentiated | 0.68 | 0.30-1.54 | 0.358 |  | 0.00 | 0-infinity | 0.996 |  | / | / | / |  | 0.00 | 0-infinity | 0.997 |
| Unknown | 0.66 | 0.43-1.03 | 0.067 |  | 0.90 | 0.20-3.92 | 0.883 |  | / | / | / |  | 0.90 | 0.21-3.96 | 0.894 |
| Subtype |  |  |  |  |  |  |  |  |  |  |  |  |  |  |  |
| HR+/HER2+ | Reference | | |  | Reference | | |  | Reference | | |  | Reference | | |
| HR+/HER2- | 1.74 | 0.90-3.38 | 0.100 |  | 1.52 | 0.21-11.1 | 0.681 |  | 1.00 | 0.49-2.01 | 0.993 |  | / | / | / |
| HR-/HER2+ | 0.66 | 0.08-5.19 | 0.690 |  | 0.00 | 0-infinity | 0.997 |  | 0.65 | 0.08-5.26 | 0.689 |  | / | / | / |
| HR-/HER2- | 6.18 | 1.90-20.09 | 0.002 |  | 14.84 | 0.93-237.94 | 0.057 |  | 1.83 | 0.55-6.06 | 0.323 |  | / | / | / |
| T stage |  |  |  |  |  |  |  |  |  |  |  |  |  |  |  |
| T1 | Reference | | |  | Reference | | |  | Reference | | |  | Reference | | |
| T2 | 1.70 | 1.38-2.1 | < 0.001 |  | 1.78 | 0.91-3.51 | 0.094 |  | 1.59 | 1.28-1.97 | <0.001 |  | / | / | / |
| Surgery |  |  |  |  |  |  |  |  |  |  |  |  |  |  |  |
| Mastectomy |  | Reference |  |  |  | Reference |  |  |  | Reference |  |  |  | Reference |  |
| Breast-conserving surgery | 0.56 | 0.45-0.69 | <0.001 |  | 0.72 | 0.36-1.41 | 0.333 |  | 0.59 | 0.48-0.73 | <0.001 |  | 0.76 | 0.38-1.49 | 0.420 |
| Chemotherapy |  |  |  |  |  |  |  |  |  |  |  |  |  |  |  |
| No/unknown | Reference | | |  | Reference | | |  | Reference | | |  | Reference | | |
| Yes | 0.37 | 0.21-0.64 | < 0.001 |  | 1.26 | 0.44-3.57 | 0.665 |  | 0.69 | 0.38-1.28 | 0.239 |  | / | / | / |

Abbreviations: OS, overall survival; BCSS, breast cancer-specific survival; HR, hazard ratio; CI, confidence internal.
